# Adenosquamous carcinoma of the pancreas: two case reports and review of the literature 

**DOI:** 10.1186/s13256-022-03610-5

**Published:** 2022-10-30

**Authors:** Daniel Paramythiotis, Filippos Kyriakidis, Eleni Karlafti, Triantafyllos Didangelos, Ilias-Marios Oikonomou, Anestis Karakatsanis, Christos Poulios, Eleni Chamalidou, Anastasios Vagionas, Antonios Michalopoulos

**Affiliations:** 1 1st Propaedeutic Surgery Department, AHEPA University General Hospital of Thessaloniki, Aristotle University of Thessaloniki, Thessaloniki, Greece; 2 1st Propaedeutic Internal Medicine Department, AHEPA University General Hospital of Thessaloniki, Aristotle University of Thessaloniki, Thessaloniki, Greece; 3 Emergency Department, AHEPA University Hospital of Thessaloniki, Aristotle University of Thessaloniki, Thessaloniki, Greece; 4 Department of Pathology, School of Health Sciences, Faculty of Medicine, Aristotle University of Thessaloniki, University Campus, bld. 17b, 54124 Thessaloniki, Greece; 5 General Hospital of Kavala, Kavala, Greece

**Keywords:** Adenosquamous, Carcinoma, Pancreas, Adenocarcinoma, Duodenum, Surgery, Case report

## Abstract

**Background:**

Among the total reported cases of pancreatic duct adenocarcinomas, around 1–2.9% are adenosquamous carcinomas of the pancreas. Due to limited data, preoperative diagnosis is a great challenge for physicians, and it is usually set post-operational, based on the pathologist report. We operated on two cases of adenosquamous carcinoma of the pancreas, which we present alongside the operation and treatment planning.

**Case report:**

A 69-year-old Caucasian female and a 63-year-old Caucasian male presented themselves with jaundice in our department. The abdomen computed tomography and magnetic resonance imaging scans revealed lesions of the pancreas. A pancreas–duodenumectomy was performed in both patients, and the post-operational histology analysis revealed adenosquamous carcinoma of the pancreas head. The patients were discharged in good condition and received further chemotherapy treatment after surgery.

**Conclusions:**

Two case reports of adenosquamous carcinoma of the pancreas are described here, which both underwent surgery resection. The limited available literature on this topic substantially limits the knowledge and guidance on treatment. A summarization of the available literature is attempted, alongside a description of possible fields of future research.

## Background

Pancreatic adenosquamous carcinoma (ASC) is an uncommon form of malignancy. Histologically, ASC has a mixed consistency including at least 30% malignant squamous cell carcinoma blended with ductal adenocarcinoma [1]. Among all exocrine pancreatic tumors, ASC counts for 0.38–10% of cases [1–3]. This large range can be attributed to the fact that some unresected tumors are classified as adenocarcinoma rather than ASC, and most of ASC are misdiagnosed as pancreatic duct adenocarcinoma (PDA) by the histopathological analysis [1, 2]. Major risk factors are age, sex, and race (white males around the seventh decade are at highest risk), tobacco and alcohol consumption, chronic pancreatitis, and genetic predispositions such as *ATM*, *BRCA2*, *p53*, and *PALB2* mutations [1, 2]. ASC of the pancreas (ASCP) usually has the same symptoms as PDA (mainly back or abdominal pain, but also diabetes, jaundice, or weight loss, depending on its relationship with the biliary tract and the level of endocrine and exocrine impairment caused). Compared with PDA, ASCP is more commonly located in the body or tail of the pancreas, however, the head is the most common location for both [1]. ASCPs tend to be larger than PDAs. Regarding composition, the squamous component is located more peripherally [4]. Necrotic tissue areas appear more commonly in ASCP, especially in the center of the lesion [4]. In immunohistochemical (IHC) analysis, several keratins (CK7, CK5/6, AE1/E3, CK1) are marked positive, whereas Cam 5.2 and CK20 are less frequent [5]. In ICH, also, *p63* (which is a useful tool in identifying squamous differentiation when acantholysis is observed) is present, *EGFR* is overexpressed, and E-cadherin is reduced or absent [6]. Similar to PDA, Carcinoembryonic antigen (CEA) and CA 19.9 are frequently positive [6]. Molecular markers correlated with antitumor drug performance such as *BCRP*, *MRP1*, *TOPO2A*, and *MGMT*, are overexpressed [2, 7]. In the literature there are a few cases in which ASCP existed concurrently or in association with intraductal papillary mucinous neoplasm [8, 9].

The two cases presented in this article are cases of this rare form of malignancy, ASCP, and because of the rarity of this disease, this article can be a valuable source of information for other medical professionals facing similar conditions. Moreover, an intriguing fact is the totally different course the two patients had following the surgery, as will be thoroughly discussed below.

## Case report

**Fig. 1:**
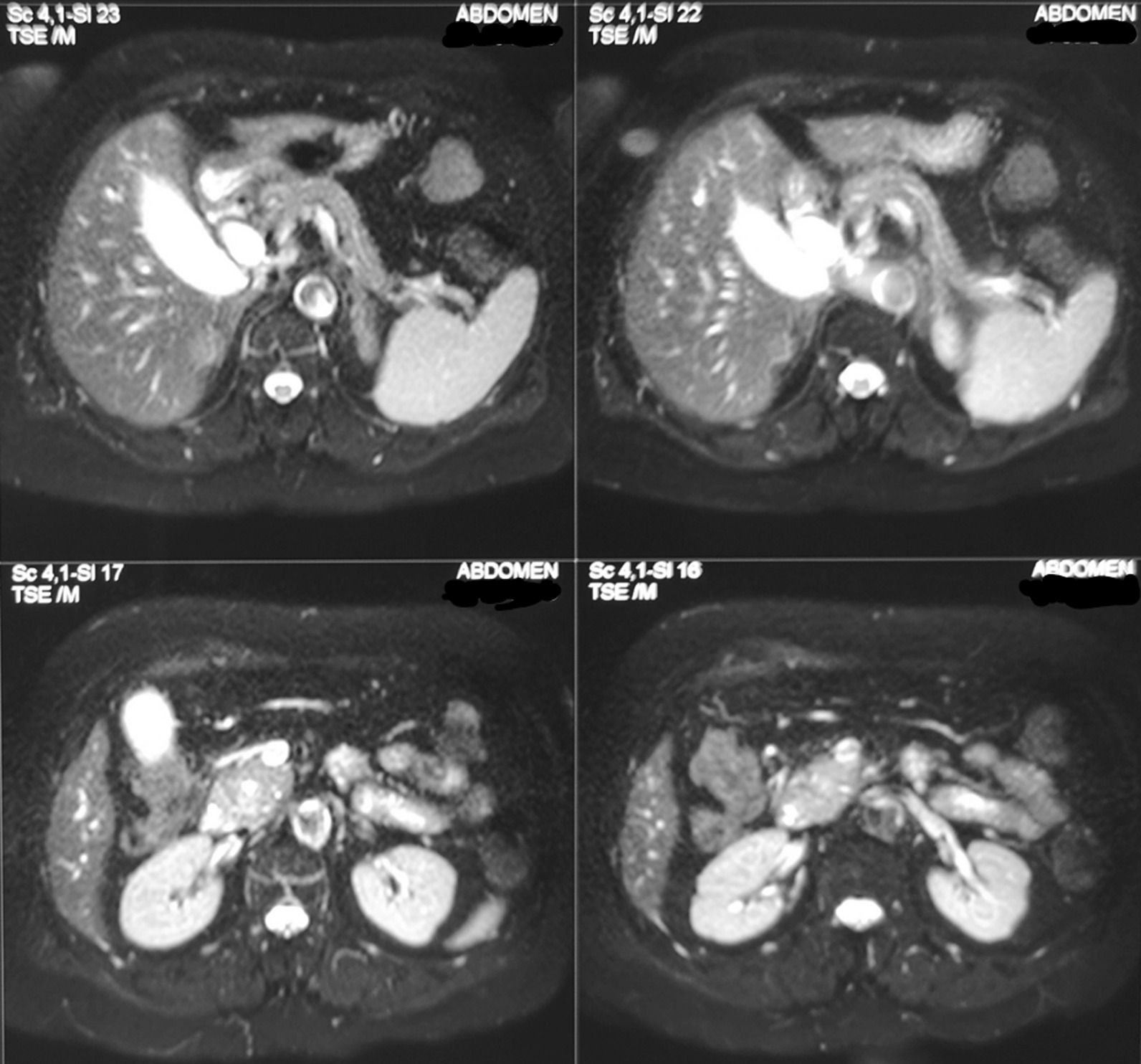
Magnetic resonance imaging image of the pancreatic tumor. Patient 1

The first patient was a 69-year-old Caucasian woman, who visited the hospital because of painless jaundice and substantial weight loss during the past months. No other symptoms, such as epigastric pain, nausea, or vomiting, were reported. She had an old medical history of an ischemic stroke years ago and hypertension for which she received *per os* acetylsalicylic acid 100 mg, furosemide 40 mg, and amlodipine 5 mg at home. She did not consume alcohol or smoke. She had one daughter, and her profession was housekeeping. During neurological examination, the patient was alert, left hemiparesis was observed (obviously due to the old stroke), sensation and reflexes were normal, and muscle tone was reduced (3−4/5) on the left upper and lower limbs and normal on the right limbs. The rest of the clinical examination did not reveal any specific findings other than the jaundice that was observed on the skin and the conjunctiva. The abdominal examination revealed no masses or tenderness. No edema was observed. The auscultation of lung and heart was normal and so was the chest X-ray. Her vital signs were as follows: blood pressure: 137/82 mmHg, pulse: 89 beats per minute (bpm), SpO2:98%, and temperature 36.6 °C. She was then scheduled for a computed tomography (CT) and magnetic resonance imaging (MRI) scan for upper and lower abdomen. The scan revealed a mass lesion in the pancreas head, about 20 mm in diameter. The lesion was inflicting outward pressure to the bile duct, causing a subsequent dilatation before the narrowing point of the duct. No metastasis was observed on the scans (Fig. 1).

The patient’s initial laboratory values are described in Table 1.

**Table 1:** First patient’s laboratory values

WBC	5490 K/ml
NE	3100 K/ml
HGb	13.5 g/dl
HCT	40.0%
PLT	210,000 K/ml
SGOT	290 U/I
SGPT	654 U/I
Total bilirubin	5.22 mg/dl
Urea	25 mg/dl
Cr	0.61 mg/dl
Glucose	99 mg/dl
INR	1.01

*WBC* White Blood Cells, *NE* Neutrophils, *HGb* Hemoglobin, *HCT* Hematocrit, *PLT* Platelets, *SGOT* Serum Glutamic Oxaloacetic Transaminase, *SGPT* Serum Glutamic Pyruvic Transaminase, *Cr* Creatinine, *INR* International Normalized Ratio

After the necessary discontinuation of the acetylsalicylic acid, a pancreas-duodenumectomy was performed, maintaining the pylorus (Longmire–Traverso operation). After surgery, for the first 4 days, 3 L of Ringer’s lactate, cefoxitin 1 g (two doses), and metronidazole 500 mg (three doses) were administered intravenously daily, and she also received 1 g of intravenous paracetamol every 6 hours and two treatments of 100 mg tramadol for mild pain at the incision site, with no further symptoms. After 4 days, according to the principles of fast-track surgery, feeding, initially only with liquid meals, and mobilization of the patient cautiously begun, and the administration of fluids was gradually reduced. The patient was discharged after 16 days in good condition. Her stay was prolonged due to a post-operational hydronephrosis, which required the placement of a ureteral pigtail-type stent.

**Fig. 2:**
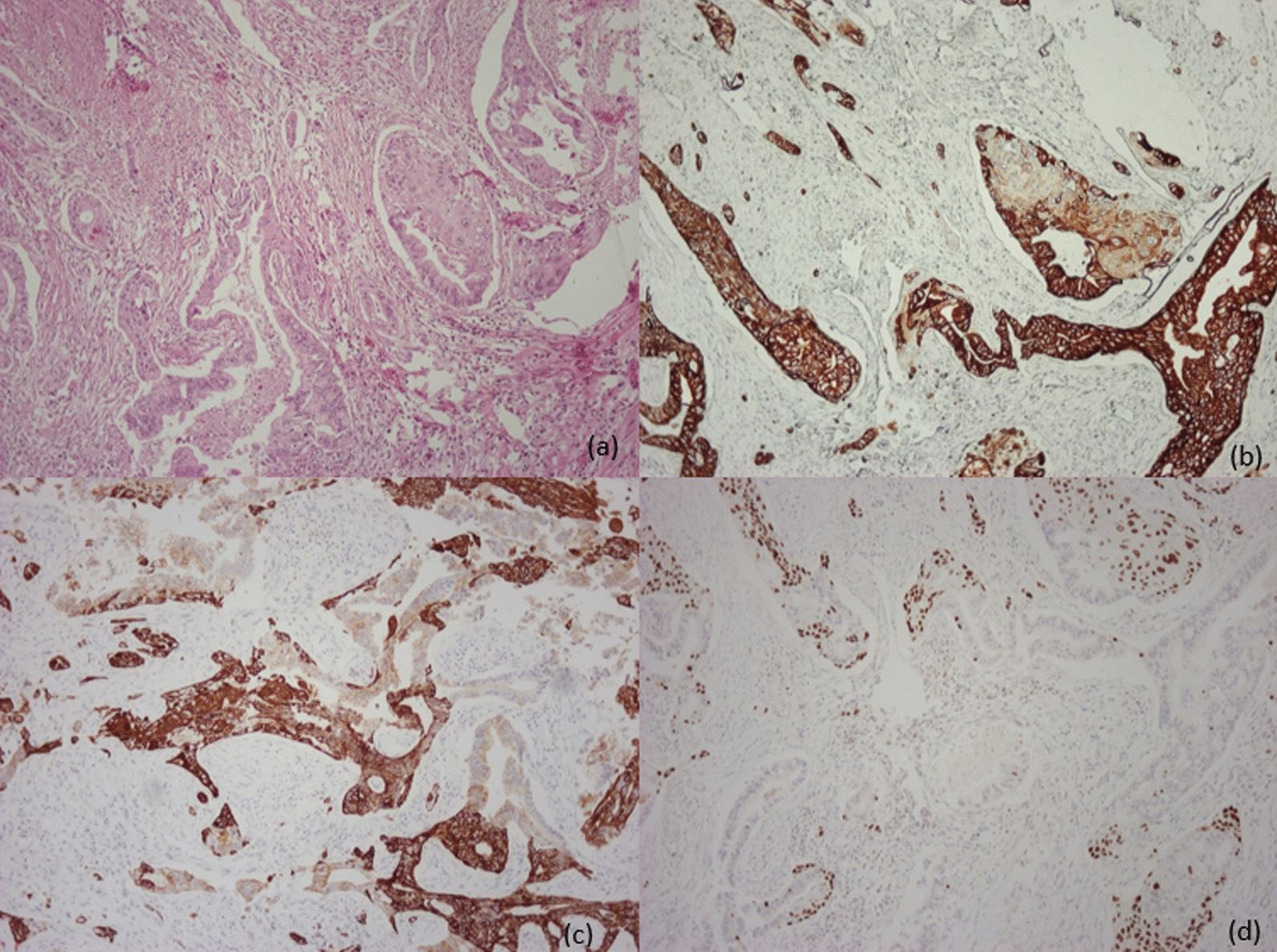
Histology samples of the ASCP. Patient 1: Stain hematoxylin/eosin (**a**) showing a carcinoma consisting of both adenomatous and squamous components. The former was positive for *CK19* and *CK7* (**b**) and the latter was positive for *34βE12* (**c**) and *p63* (**d**). All images × 100 magnification.

The pathologist’s report described an ASC of the pancreas head of pT3N0, according to TNM staging, and elements of this report are summarized on the image bellow (Fig. 2). Also worth mentioning is that the surgical resection was R0.

The patient’s treatment continued with chemotherapy (toxicity-stop) and actinotherapy. The plan for the adjuvant treatment was a scheme including irinotecan (180 mg/m^2^), oxaliplatin (85 mg/m^2^), leucovorin (400 mg/m^2^), and 5-fluorouracil (400 mg/m^2^ as a bolus over 2 hours on day 1, then 2400 mg/m^2^ over 46 hours). Unfortunately, chemotherapy treatment was abandoned during first administration, due to toxicity that led to a short admission and inpatient treatment. Actinotherapy was resumed with no further complications. Her tumor markers as measured during follow-ups are described in Table 2.

**Table 2:** First patient’s tumor markers

Five months after surgery	
CEA	1.31 ng/ml
CA 19-9	193.8 U/ml
Eighteen months after surgery	
CEA	3.53 ng/ml
CA 19-9	142 U/ml

*CEA* Carcinoembryonic antigen, *CA 19-9* Cancer antigen 19-9

The patient remained relatively healthy for 18 months; she passed away 20 months post-operation due to cardio-respiratory failure.

The second patient was a 63-year-old Caucasian male, who sought expert consultation due to painless jaundice and recently developed diabetes mellitus. The diabetes was diagnosed about 3 months ago and he was taking 1000 mg metformin *per os*, once daily at home. He had no other medical history and received no further medication at home. Regarding alcohol, he was a social user and smoked at a total level of 5 pack years. The patient had one daughter and his profession was a bus driver. The clinical examination revealed a mild epigastric abdominal pain. No other symptoms, such as malaise, nausea, or vomiting, were reported. McBurney’s sign, Rovsing’s sign, Blumberg’s sign, and, interestingly, Courvoisier’s sign were all negative. No edema was observed. The auscultation of lung and heart revealed nothing worth mentioning, and the chest X-ray was normal. During neurological examination, the patient was alert, all cranial nerves functioned well, and muscle tone, reflexes, and sensation were normal. His vital signs were as follows: blood pressure: 145/87 mmHg, pulse 84 bpm, SpO2: 98%, and temperature 36.7 °C. According to these findings and taking into consideration the recently developed diabetes, he was examined with a CT and MRI scan for upper and lower abdomen to exclude or reveal the presence of a pancreatic tumor. This revealed a 34 mm lesion on the pancreas head, in conjunction with the superior mesenteric and gastroduodenal artery. The scan revealed no metastatic lesions. A fluorodeoxyglucose-positron emission tomography (FDG-PET) scan was considered as an additional option, even though it is not a necessity, but cost of this examination is not covered by the state for cases of pancreatic cancer and the patient could not afford the cost himself. Thus, the staging was performed with the methods mentioned above.

The patient’s initial laboratory values are described in Table 3.

**Table 3:** Second patient’s laboratory values

WBC	8700 K/ml
NE	6500 K/ml
HGb	12.7 g/dl
HCT	36.7%
PLT	261,000 K/ml
SGOT	440 U/I
SGPT	718 U/I
Urea	14 mg/dl
Cr	0.70 mg/dl
Total bilirubin	25.17 mg/dl
Direct bilirubin	21.93 mg/dl
Glucose	241 mg/dl

*WBC* White Blood Cells,* NE* Neutrophils,* HGb* Hemoglobin,* HCT* Hematocrit,* PLT* Platelets,* SGOT* Serum Glutamic Oxaloacetic Transaminase,* SGPT* Serum Glutamic Pyruvic Transaminase,* Cr* Creatinine

The patient had to be initially treated with chemotherapy, in a scheme including irinotecan (180 mg/m^2^), oxaliplatin (85 mg/m^2^), leucovorin (400 mg/m^2^), and 5-fluorouracil (400 mg/m^2^ as a bolus over 2 hours on day 1, then 2400 mg/m^2^ over 46 hours). The patient’s tumor markers, as measured during the neoadjuvant treatment, are described in Table 4.

**Table 4:** Second patient’s tumor markers

Three months before surgery	
CEA	8.44 ng/ml
CA 19-9	179.2 U/ml
One month before surgery	
CEA	10.32 ng/ml
CA 19-9	69.24 U/ml

*CEA* Carcinoembryonic antigen,* CA 19-9* Cancer antigen 19-9

This was followed by a pancreas-duodenumectomy, maintaining the pylorus (Longmire–Traverso operation). After surgery, for the first 4 days, 3 L of Ringer’s lactate, ciprofloxacin 400 mg (two doses), and Metronidazole 500 mg (three doses) were administered intravenously daily, and he also received 1 g of intravenous paracetamol every 6 hours and two doses of tramadol 100 mg for mild pain at the incision site, with no further symptoms. After 4 days, feeding, initially only with liquid meals, and mobilization of the patient slowly begun, and the administration of fluids was gradually reduced. He was discharged in good condition on the eight post-operational day.

The pathologist report revealed ASC of the pancreas head of pT2N1, in accordance with TNM staging. According to the report, the squamous element was more than 30% of the total carcinoma. Markers of squamous differentiation such as *CK5/6*, *CK34BE12*, and *p63* are positive on the squamous element and mildly expressed or absent on the ductal adenocarcinoma element. The tumor was 2.7 cm in the greatest dimension and so was classified as T2, and metastasis was detected in one regional lymph node and thus it was classified as N1, according to TNM staging. Also, worth mentioning is that the surgical resection was R0.

**Fig. 3:**
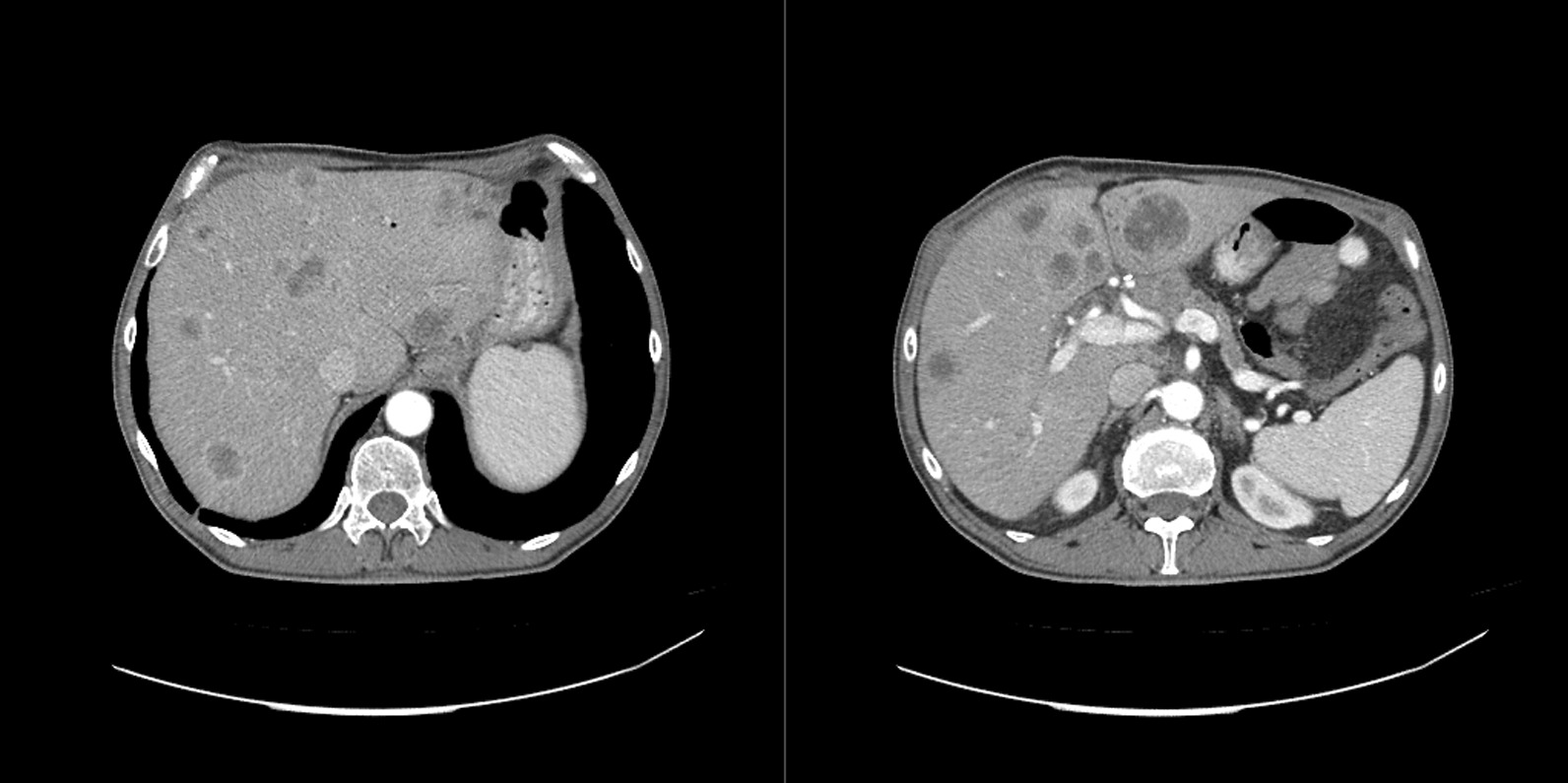
Magnetic resonance imaging image of the liver metastatic lesions. Patient 2

The further chemotherapy treatment consisted of gemcitabine (1000 mg/m^2^) and paclitaxel (100 mg/m^2^). Six months post-operation, a scheduled abdomen scan revealed multiple metastatic lesions in the liver (Fig. 3). The patient was under treatment and in a general good condition, but a few months later he died after a stroke.

## Discussion

Two cases of ASCP have been presented and since data are scarce for rare conditions, cases such as these may help medical professionals understand and treat this disease better. What is more interesting is the completely different course of the two patients. First and foremost, both patients had an excellent surgery, with R0 resection and no complications (the role of surgery is crucial both in terms of survival and diagnosis since the simple tissue biopsy may misclassify the condition). The first patient received almost no chemotherapy, had radiotherapy after surgery, lived for 20 months after surgery with a good quality of life, and died probably from a cause unrelated to her tumor. On the other hand, the second patient received chemotherapy in a neoadjuvant and adjuvant setting and, despite the R0 resection, had several liver metastases only 6 months after surgery, and died a few months later. In this section, we discuss that there is no proof that chemotherapy is inferior to radiotherapy and thus the different course of these patients remains a mystery. It is hard to tell if the second patient had micro-metastasis, not visible, from the beginning and that is the reason for his poor response to treatment, but this could be answered only with PET, and even then might not be. However, this demonstrates the complexity of this rare form of malignancy and the importance of such cases to science.

The pathophysiology of ASCP remains poorly understood. The most intriguing question is how the squamous element is created. Autopsy reports of normal pancreas show a metaplasia into squamous epithelium in 17–48% of cases, despite the fact that squamous differentiation does not exist in normal pancreatic tissue. [2, 10]. Squamous metaplasia can appear in cases of chronic pancreatitis and after stent placement in the main pancreatic duct [11, 12], as well as in the wall of benign pancreatic cysts [13].

To explain this mystery several theories have been proposed:

1. Chronic pancreatitis or obstruction of the pancreatic duct by a tumor, as causal factors of chronic inflammation, result into squamous metaplasia of the ductal cells, and then into conversion to ASCP [5, 14].
2. There is also a collision theory proposing that formation of ASCP is caused by the combination of two histologically different and functionally independent neoplastic cell populations in the pancreas [14–16]. The same KRAS mutation has been observed by a few studies in both tumor lines [6]. This may imply a common origin, which reduces the likelihood of the collision hypothesis.
3. According to the third theory, there are multipotent primitive cells, some of which are differentiated into adenocarcinoma and others into squamous cell carcinoma (SCC), leading to a tumor that contains both cell types [3, 14].

Unlike PDA, that collapse of the vasculature and a low microvascular density are typical, in ASCP the data about angiogenesis are scarce. A single case report is the source on this matter [17]. In this report, in comparison with a PDA case, in the ASCP case elevated numbers of tryptase-positive mast cells (MCs) and microvascular density were observed. Notable angiogenic activity in ASCP is demonstrated by these data, with a speculated role of miR-27a-3p, miR-21-5p, miR-122-5p, and miR-181a-5p in the adjustment of this procedure [17].

As far as it concerns the expression of miRNAs and genes, data shows that the angiogenetic pathways were more active in ASCP with respect to PDA, and morphometric evaluation confirmed the evidence of a higher number of microvessels in ASCP compared with PDA, further supporting these data [17].

Overexpression of Ang-2 seems to have a correlation with bad prognosis in various tumors [18, 19]. Ang-2 is a competitive inhibitor of Ang-1 regarding its binding to the receptor Tie-2, enhancing vascular endothelial growth factor (VEGF)-mediated angiogenesis, since it has been shown that VEGF as well as tissue hypoxia upregulate the expression of Ang-2. A higher blood supply in ASCP is suggested by the persistence in the portal vein phase, as observed in enhancement contrast pattern on CT [20]. MCs have an important role in the formation of new blood vessels inside the tumor, through the release of an active serine protease, tryptase, which is the most significant angiogenic factor of these cells [21]. Another typical feature of PDA is the existence of a fierce fibro-inflammatory reaction, which is absent in ASCP due to the tryptase that cut regimens such as fibronectin, thus favoring angiogenesis [17]. The worse outcome of ASCP can partially be explained by the crucial role of angiogenesis in cancer growth and metastasis [17]. Furthermore, the verification of these findings might suggest a potential role of anti-VEGF factors such as bevacizumab in the treatment of ASCP.

Concerning its genetics, sporadic mutations are the cause for the majority of pancreatic cancers, whereas inherited germline mutations are considered to be responsible for only 10% [22].

Immunohistochemistry has demonstrated a high expression of cytidine deaminase (CDA) within the glandulous and, to a lesser extent, the undifferentiated elements of ASCP [23]. CDA contributes to gemcitabine being inactivated [24] and capecitabine being activated [25]. In two studies, a higher response to treatment with gemcitabine in pancreatic cancer patients was correlated with low circulating CDA activity [26, 27], however, the results were not the same in a later multicenter prospective trial in which 120 patients received treatment with gemcitabine [28]. Preclinical data concerning intratumoral CDA expression have shown high levels of expression to be correlated with decreased response to gemcitabine [29] and a higher response to capecitabine [30]. These data support the assumption that CDA status may have a very important role in selecting the right agent between capecitabine and gemcitabine.

A highly elevated risk of carcinoma of the pancreas has been noticed in germline *CDKN2A* mutation carriers [4]. The molecular alterations in ASCP resemble those detected in PDA [6], being the loss of p16 (which is the protein that the gene *CDKN2A* encodes) a usual process in the first steps of the development of pancreatic cancer. As a typical example, in kindred with familial atypical multiple mole melanoma, the risk of pancreatic cancer, in the carriers of a germline *CDKN2A* mutation, is 38 times greater than in the general population, according to estimations [22].

Furthermore, the *UGT1A1* gene is accountable for the metabolism of SN-38, which is an active metabolite of irinotecan, and different variations of *UGT1A1* (such as UGT1A1*6 and UGT1A1*28) increase myelosuppression, such as serious neutropenia [31]. Thus, it is a possible factor to be tested in case of irinotecan usage.

Diagnosis for ASCP is based on endoscopic ultrasound (EUS) biopsy and CT imaging. ASCP usually presents in the CT as a round or lobulated mass [32] and frequently shows peripheral contrast enhancement in the arterial phase, which remains in the venous phase [20]. Moreover, it is worth mentioning that thrombosis in the portal vein system is a usual finding [32].

In the EUS, ASCP often appears as a hypoechoic and solid mass, not sufficiently defined [33]. Recent studies suggest that in a EUS-guided biopsy, conventional needles have shown inferior diagnostic performance compared with ProCore biopsy needles [34].

It is of paramount importance to point out that imaging should not only be considered as a diagnostic tool, but also as an important part in screening procedures of special populations with greatly elevated risk of pancreatic cancer, mostly with MRI or EUS [35–37].

Surgical resection constitutes the only potentially curative treatment, offering a 20% chance of 5-year survival, but only 15–20% of patients fulfill the criteria for the surgery because the vast majority have either locally advanced disease or distant metastases at diagnosis [38]. Thus, other forms of treatment are also commonly considered, either to enhance the effect of surgery or to substitute surgery when it is not possible.

The necessity of treatment with adjuvant chemotherapy after excision of ASCP is not clarified yet [39]. Taking into account the evidence base for the treatment options of the larger group of pancreatic exocrine malignancies, the use of chemotherapy in an adjuvant setting is abetted by retrospective analyses, commonly with 5-fluorouracil (5-FU) or gemcitabine as a single agent [38, 40].

A lot of combinations have been used in chemotherapy, including the following examples:

1. Chemotherapy using the FOLFU-CDDP regimen (5-fluorouracil 400 mg/m^2^ as a bolus over 2 hours on day 1, then 2400 mg/m^2^ over 46 hours plus cis-platin 50 mg/m^2^ on day 1 [41]. Compared with FolFOxIri (FX), a combination of 5-FU, leucovorin, oxaliplatin, and irinotecan, mFX (a modified analog, without the bolus 5-FU) might lead to fewer Grade 3 or 4 nonhematological adverse events (AEs), with an almost similar response rate. Nonetheless, to reduce hematological AEs, more attempts and efforts might be needed [42].
2. A common chemotherapy plan is adjuvant gemcitabine (1000 mg/m^2^ on days 1, 8, and 15 of a 28-day cycle) [23]. The combination therapy with capecitabine and gemcitabine could lead to a further survival benefit compared with single agent agent, as suggested by data from the ESPAC-4 trial [43]. Combination therapy with platinum agents may have the biggest response rates according to suggestions from retrospective analyses restricted to ASCP [44, 45]. Even though the combination of gemcitabine with platinum-based agents for ASCP was in theory more hopeful, its efficacy is only supported by some case reports [44]. *BRCA1/2* mutations observed in ASCP suggest a probable higher sensitivity to gemcitabine combined with a platinum agent [46].

Combination of 5-FU with irinotecan or cisplatin, or gemcitabine combined with 5-FU or carboplatin are the regimens included in metastatic disease chemotherapy [2].

Lastly, following an ASCP resection, the TNM 8th staging system, radiotherapy and chemotherapy may be indicators for survival benefit [47]. In one study, the chemoradiotherapy group had much better prognosi compared with the group that received neither radiotherapy nor chemotherapy, as demonstrated by the Kaplan–Meier curves. Moreover, the combination of chemo- and radiotherapy had an even greater survival outcome than the other two groups [12]. In the same study it was also found that, even though the survival in the chemoradiotherapy group was higher compared with the single therapy group, there was not any statistically significant difference in survival between chemo- and radiotherapy subgroups [47].

## Conclusion

ASCP is rarely reported, therefore there are only scarce data on its management and treatment. This reveals several problems and potential traps, including but not limited to:

1. Possible false staging of the disease in patients where, due to incomplete screening, a metastatic disease might have been misclassified as early-stage disease, thus altering the reported treatment outcome [48];
2. Small sample studies or case reports are the main source of information about the clinical features of ASCP because ASCP cases are extremely rare in comparison with PDA.
3. Misclassification of pathological specimens as ASCP by pathologists is probable due to the rarity of the disease [48].
4. There is the possibility of misclassification of an ASCP as a squamous cell carcinoma of the pancreas (SCCP) in patients that were only biopsied, as indicated by the decreased resection rate of SCCP [48].

After resection, the median overall survival (OS) of ASCP patients is 12 months, while the median OS of PDA patients is 16 months, which constitutes a statistically significant difference [1]. SCCP appears to be a more aggressive disease, both clinically and pathologically, which is less often treated with any definitive local therapy or surgically removed [48]. Furthermore, the survival data shows a worsening in the condition from PDA to ASCP and then to SCCP, which might suggest that the squamous element is worsening the prognosis, possibly due to the limited knowledge we have around SCCP and its pathophysiology. Whether the explanation is that ASCP is a more aggressive cancer or if it is our limited experience in ASCP management compared with PDA needs further research to be answered.

## Data Availability

Pub med was used as a source of information using the search term: Adenosquamous AND carcinoma AND pancreas.

## Abbreviations

ASC: Adenosquamous carcinoma
ASCP: Adenosquamous carcinoma of the pancreas
CDA: Cytidine deaminase
EUS: Endoscopic ultrasound
MC: Mast cell
PDA: Pancreatic duct adenocarcinoma
SCCP: Squamous cell carcinoma of the pancreas
